# Exploring the contributing factors of fast food consumption in daily life: a qualitative study of Saudi university students

**DOI:** 10.1186/s12889-025-23624-0

**Published:** 2025-07-07

**Authors:** Mohammed Mahmoud Sarhan, Shihanah Eid Alotaibi, Nuorah Awadh Alharbi, Shemaa Adel Aljohani, Yara Ahmad Alnazzawi, Maram Ali M. Alwadi

**Affiliations:** 1 Department of Preventive Dental sciences, College of Dentistry, Taibah University, Al- Madinah Al- Munawwarah, 42353 Saudi Arabia; 2 College of Dentistry and Hospital, Taibah University, Al- Madinah Al- Munawwarah, 42353 Saudi Arabia; 3 Department of Dental Health, College of Applied Medical Sciences, King Saud University, P.O. BOX 145111, Riyadh, 4545 Saudi Arabia

**Keywords:** Fast food, Factors, Consumption, Public health

## Abstract

**Background:**

Fast food is being increasingly consumed in the modern world. This raises urgent questions about its impact on our health. There is a large body of research examining the factors contributing to fast food consumption but few studies have been conducted in Saudi Arabia among university students. Moreover, there is almost a total lack of qualitative studies in this area to explore the details and contexts of these factors. This study aims to address this gap by conducting a qualitative investigation into the factors associated with fast food consumption in the daily lives of Saudi college students.

**Methods:**

The subjects of this qualitative study were 22 students from a university in Al Madinah Province, the Kingdom of Saudi Arabia (KSA). Data was collected over 10 weeks through diary entries and semi-structured interviews, which were held virtually. Thematic analysis was then conducted on the data, with an inductive strategy used to identify emerging themes.

**Results:**

The factors previously found to contribute to fast food consumption, such as taste and convenience (time, effort, and money-saving qualities) were confirmed in this study. Moreover, the research found that the growing social narrative around fitness and health has led to a rise in the public’s concerns about health, which, in turn, has prompted many people to think again about their consumption of fast food. This study also reveals the effect of culture on fast food consumption, illuminating interesting perspectives on the lack of this consumption in various ritual and cultural situations.

**Conclusion:**

The results of this qualitative study reveal important findings about the contributing factors associated with fast food consumption among the under-researched study population of university students. The contributing factors identified are taste, convenience, health concerns, and cultural influences. By developing our understanding of these factors, such as the details of their contextuality and their impact on people’s eating behaviors, we can develop more effective public health interventions. Both health practitioners and policymakers can then use this comprehensive understanding of these factors to create interventions that respond to people’s cultural contexts and personal preferences, enhancing health outcomes for the public.

## Background

In the modern world, fast food now forms an important part of people’s daily lives and is among the fastest-growing food categories worldwide [1]. “Fast food,” per the Cambridge Dictionary (2022), comprises meals that are already cooked or are quickly prepared and served rapidly [2]. A large volume of research has identified a persistent connection between negative nutritional outcomes and frequent fast-food consumption [3–6]. One study, for instance, highlights the significance of the amount of energy, saturated fat, and other fat found in fast food to poor nutritional outcomes, noting that these foods are consumed in the place of healthier options such as fruit and milk and are consumed alongside large volumes of high-sugar drinks [3]. Further research by Barnes et al. [5] similarly finds a link between fast food and poor nutritional intake, showing that higher consumption of fast food is associated with increased energy consumption and decreased intake of fiber, as well as decreased fruit and vegetable consumption.

The link between poor health outcomes and fast food consumption is a frequent theme of the findings in this field [7–11]. The 2005 longitudinal research by Pereira et al. [10] on the effects of fast food consumption finds that people who eat fast food more than twice weekly are 4.5 kg heavier and their insulin resistance is 104% higher than their non-fast-food-eating counterparts, increasing their risk of obesity and type 2 diabetes. Bodicoat et al. [8] similarly identify a connection between an increased risk of obesity and type 2 diabetes and the availability of fast food outlets. Research in the area of dentistry emphasizes the link between the development of dental caries and the consumption of fast food [12, 13], suggesting that the development of dental caries is associated with the increased consumption of sugar.

Even though research repeatedly identifies a link between poor health and fast food, it equally finds that fast food consumption is rampant in a variety of age groups [14–18]. In the US, over a third (36%) of children and adolescents eat fast food every day [14]. In the context of the Kingdom of Saudi Arabia (KSA), studies reveal that 86.7% of the population overall consumes junk or fast food [15]. This research looked at a cohort of 355 Saudi Arabian individuals, most of whom (61.4%) were single, most (63.9%) were educated with a university or higher degree, and the slim majority (56.3%) were aged 20 to 40 years old. Consumption of fast food was found to be lowest among the oldest participants surveyed, who were aged 40 to 50, and highest among the youngest individuals surveyed, aged from 10 to 20 [15]. Research conducted in Jeddah on 239 individuals reported similar findings, with the vast majority (85%) of Saudi Arabian adolescents stating that they would rather eat fast food than a homemade meal [16]. This finding was reinforced by the results of a cross-sectional study among women and girls in Riyadh, specifically, 127 school girls aged 19–29 from Princess Nourah Bint Abdulrahman University middle and secondary school and 69 college students from 19 to 29 years of age. According to this research, almost all (95.4%) of those surveyed ate fast food and most (89.1%) did so one or more times every week [17].

Worldwide, research has uncovered several contributing factors to fast food consumption across different demographics. A key driver of this consumption generally is taste [19–21]. Promotional offers and cost-effectiveness are also frequently identified as contributing factors [19–23]. The research also finds that the time and effort-saving nature of fast food, that is, its convenience and easy accessibility, is a strong driver of its consumption [20–24]. Among adolescents, social factors such as familial roles and peer pressure substantially influence the choice to consume fast food [19, 21, 25]. Moreover, our positive opinions of fast food are driven by advertising and the media [19–22]. However, the contributing factors of fast-food consumption in the KSA specifically have been the subject of only a relatively few researchers [17, 18, 26, 27]. Of the few studies available, one conducted in Riyadh by Alfaris et al. [17] on Saudi Arabian adolescents and young women found that the leading contributing factors of fast food consumption are taste and convenience. More recent research on college students in Majmaah by AlFahied et al. [26] emphasizes the role of taste and advertising in driving fast food consumption. Additionally, limited time, changes in daily routines, education, and taste were identified as key influencing factors of the consumption of junk food among adults in the Saudi Arabian city of Jeddah in research by Mandoura et al. [18], and Althubaiti [27] found that the key influencers of diet choices were little awareness of health issues, busy daily lives, the elevated cost of vegetables and fruit, and taste among a study population of students at Umm Al-Qura University.

While the existing research provides useful data on fast food consumption overall, only a few studies have focused on university students in the KSA, particularly in Al Madinah Province. Additionally, most existing studies are quantitative. Although they are valuable, they do not provide enough detailed knowledge on this topic. Therefore, a qualitative approach examining the factors associated with fast food consumption among university students will provide this detail and enrich the existing literature. Consequently, this research will explore fast food consumption in the daily lives of university students in the KSA to increase our understanding of this issue for the benefit of healthcare practitioners, policymakers, and other stakeholders. The results of this research will inform the development of more effective health interventions and healthy eating strategies for application among Saudi university students and, potentially, the wider public.

## Methods

To explore the factors that contribute to fast food consumption, a qualitative research methodology was used in this study to both collect and analyze the data.

### Study context

This research enlisted students attending university in Al Madinah Province, KSA, to participate in this research. The participants were all undergraduates at the same government university located in the province. Al Madinah Province, also known as the Madinah Region, is an area of the KSA containing 13 territories [28]. The region is located along the Red Sea coast in the nation’s west and its capital city is Al Madinah or Medina [28]. The region spreads over 149,207 sq.km. and is home to 2.1 million people as of 2017 [28]. Al Madinah Province’s education system is overseen primarily by the Ministry of Education [29], and around 724 boys and 733 girls attend school in the area. Taibah High School is one of the more highly reputed schools in the region [29], and several higher education facilities are located in the Province. Taibah University is a notable center for further education in the area and it boasts 16 colleges dotted throughout Al Madinah Province [30]. Founded in 1989, Industrial College is also a significant higher education facility and has a student body consisting not only of students across the KSA but also across the globe [29]. The Al Madinah College of Technology established in 1998 is a further notable Al Madinah educational institution [31]. It offers a variety of STEM degrees such as computer technology, mechanical technology, electrical technology, administrative technology, and electronic technology [31]. This region is a pioneer in education in the country and provides an environment rich in culture, making it a significant contributor to the ongoing growth of the KSA [29].

### Sampling techniques

Both purposive and snowball sampling were used in this study. Per purposive sampling, participants were chosen among undergraduate students completing a bachelor’s degree at a specific university in Al Madinah Province, KSA, who consume fast food at least once a week.

The research team conducted the participant recruitment. Specifically, they visited the university’s colleges at random where students gathered at break times. Once they identified a potential participant, they then approached the student, explained the research, and invited them to join the study. If the participant met the inclusion criteria and was willing to participate voluntarily, the research team collected their contact details to coordinate the data collection.

Following purposive sampling, snowball sampling was then used. This took place during the data collection process where the first group of participants were asked to suggest other potential participants from among their fellow university students. The contact details of these potential participants were given to the research team who contacted the students and checked they met the inclusion criteria and were willing to participate in the study voluntarily. Using snowball sampling alongside purposive sampling in this research facilitated the recruitment of relevant participants who could address the research aim on factors associated with fast food consumption.

As per Ritchie and Spencer [32], data saturation was reached when no new data or themes were revealed by subsequent interviewees, which occurred when data had been gathered from 22 participants. There was thus no reason to recruit further participants to the study after this point.

### Study participants

A total of 22 university students, 8 males and 14 females, participated in this research. All participants were aged 20 to 25 and were from one of 6 different colleges of the same university. In total, 8 participants were interviewed, and 14 completed diary entries. Further characteristics of the interviewed participants and those who completed diary entries are summarized in Table 1.

**Table 1:** Characteristics of interviewed participants and those who completed diary entries

Characteristic	Interviewed (*n* = 8)	Diary Entries (*n* = 14)
**Gender**		
Male	3	5
Female	5	9
**Age range (Years)**	20–25	20–25
**College**		
Dentistry	2	1
Medicine	2	4
Pharmacy	1	0
Family Sciences	0	3
Computer Science and Engineering	2	3
Science	1	3

### Data collection methods and instruments

Two data collection methods were used in this research, namely, diary entries and semi-structured interviews, which were conducted virtually. All data collection took place over 10 weeks from September to November 2023. The participants chose which data collection method they preferred, either diary or interview. The researchers allowed the participants to make this choice to ensure they were comfortable sharing their stories and information to try and elicit more accurate, detailed data. For example, a shyer participant may prefer to write down their thoughts rather than discuss them and thus the diary would draw out more information from this participant.

Additionally, all the participants who chose to be interviewed were given the option to attend an in-person interview or a virtual interview. All of them chose to participate in a virtual rather than in-person interview. Arabic was the primary language used in the interviews, but the participants could choose to speak in English if they wished. Again, the choice of language and in-person or virtual interviews were given to participants to increase their comfort levels and thus the likelihood that they would provide accurate, detailed information. The interviews lasted an average of half an hour. Before the interviews, each participant received the interview questions as a Word document via WhatsApp so they could reflect on the questions and note down anything they wished to discuss in the interview. The researchers took this step to improve the data collection process and minimize the potential for participants to forget a point they wished to mention. The participants and the research team also used WhatsApp to coordinate the times of the interviews. The interviews were conducted using Zoom or Microsoft Teams based on the participants’ preferences.

All of the participants who chose to complete a diary asked to receive the prompts for their entries in Arabic and subsequently wrote their diary entries in Arabic. The research team used the interview questions as the diary entry prompts, writing them in a Word document and sending the document to the participants using WhatsApp, their preferred contact method. The participants took several days to complete their diary entries and return them to the researchers. When writing their diaries, some of the participants contacted the researchers to ask them to expand on some points and the researchers guided them and answered their questions.

The researchers used their academic experience and drew on relevant existing literature to prepare the questions for the diaries and interviews. All questions were open-ended so that the participants could discuss any issues and experiences they felt were relevant. The questions were as follows:

1. What is your daily routine in the week, on the weekend, and during vacations, and how do meals fit in?
2. Please tell us more about your mealtime routines.
3. Please give us some examples of situations that drove you to eat fast food or stopped you from eating it.
4. What are your reasons for eating fast food?
5. What does eating fast food mean for you?

During the interviews, where necessary, the participants were encouraged to give more details about their answers by the interviewer who would ask for more examples or more detailed explanations. The insights gained from the early interviews were used in later interviews to improve the interview questions and identify new avenues of questioning.

When the participants completed their diaries and returned them to the research team, the team reviewed the diaries and, again where necessary, asked the participants to, for example, elaborate on certain points and give additional examples to provide fuller information.

Audio recordings were taken of all the interviews to ensure that all the information was captured accurately.

### Data processing

As soon as possible following each interview, the research team took the audio recording from the interview and transcribed it. Then, they translated the transcription from Arabic into English. The research team was responsible of the translation. All of the members of the research team are native Arabic speakers but most have a largely English-language academic background and one member of the team is a native speaker of both Arabic and English. The research team completed the translation process across a series of meetings. For the diaries, once they were completed and returned by the participants, they went through the same translation process. The reason for translating the data as it was collected was to start the data analysis with the first lot of data gathered.

### Data analysis

The approach to the data analysis adopted in this study was agreed upon by the researchers and began with the first interview. The researchers chose to use thematic analysis and followed the steps for thematic analysis laid out by Braun and Clarke [33–35]. Specifically, the researchers first familiarized themselves with the data before then creating the initial data codes. Next, the themes were identified. They were subsequently reviewed, refined, and finalized. Lastly, a written report discussing the study results was created.

Of the seven members of the research team, six participated in the data analysis, which was led by the first author. To begin the data analysis, the translated transcriptions of the first interview and diary were reviewed. The research team worked together to help bolster the data analysis’ validity, credibility, and accuracy. Regular team meetings including the first author and a minimum of three other research team members were held to complete the data analysis.

The research team started the data analysis process by first exploring the collected data and reviewing the translated transcripts of the interviews and diaries in detail. In this way, the team was able to develop a more profound comprehension of the data gathered. Given that both the diaries and interviews were available in written form and designed to fulfill the same research objective (to explore the factors influencing fast food consumption), all of the interview and diary transcripts underwent the same analytical process.

When reviewing the data, the researchers became increasingly familiar with the patterns that were emerging from the information gathered. During the review process, the researchers took note of important findings and any first thoughts on the structures that the data was revealing. As the data analysis process progressed, the research team started to generate the initial data codes. Next, the researchers analyzed the translated interview and diary transcripts line by line, giving any relevant extracts the appropriate code. The codes were used to categorize the data and were descriptive in nature. After the first data codes were generated, the researchers used them to identify the data’s key themes. They did this by reviewing the coded extracts to find repeating ideas within the transcripts, thus revealing the data’s potential themes.

The different data themes were identified at different times throughout the course of the data analysis. Analysis began with the first transcripts and continued as additional transcripts were created and translated. No new themes were believed to be identified after the review of the transcription of the data collected from the 22nd participants, meaning that data saturation had been reached and all relevant themes had been found.

Once the potential themes were identified, the next step was for the researchers to review these themes and refine them. This was done to ensure that the themes accurately reflected the data.

When the themes had been reviewed and refined, the research team gave them suitable descriptive names. The purpose of the names was to accurately describe what each theme was about and its core idea. After the themes were finalized and their naming was complete, the research team wrote up the results section of the research report where they expanded upon the themes and created a comprehensive narrative to explain them. Preliminary data in the form of transcript quotes were used to support the explanations of the themes, provide real-world examples, and strengthen the analysis’ credibility. The researchers chose to complete the data analysis in this qualitative study using a paper-based, manual approach and thus did not use any computer software in the analysis.

Braun and Clarke [33] argue that researchers must clarify several key points before embarking on thematic analysis. Specifically, they must define what constitutes a theme, determine if they should apply inductive or theoretical analysis, decide whether realist or constructionist thematic analysis is most suitable, and determine if their themes are semantic or latent.

The themes this research identifies are believed to reflect the meanings of the collected data, as per Braun and Clarke [33]. However, also as described by Braun and Clarke [33] and Nowell et al. [36], how frequently a theme arises in the data is not considered to accurately reflect its significance. Thematic analysis is applied in this study whereby the themes are not pre-prepared based on the review of existing theories. Rather, themes are taken directly from the data collected from the study participants. Thus, this research adopts a realist thematic approach as it is believed that the data collected from the study participants is a true reflection of their experiences and not simply the result of social or cultural pressures. Furthermore, as the themes identified reflect the analysis of the data’s direct meanings, this study’s themes are semantic. The researchers did not examine elements outside of the direct meanings of the participants’ responses, such as the social and cultural pressures that could have affected these responses.

### Strategies to confirm the data analysis’ trustworthiness and credibility

Triangulation techniques and an audit trail were used to confirm the data analysis’ trustworthiness and credibility. Triangulation involved collecting data from two data sources (i.e. diaries and semi-structured interviews). Moreover, the researchers’ data collection and analysis processes and decision-making were recorded through an audit trail.

### Ethical considerations

The privacy and rights of the study participants were protected throughout this research. Ethical approval for the study was provided by the Institutional Review Board of Taibah University (TUCDREC/O20323). In particular, participant confidentiality and anonymity were safeguarded at all stages of the study. Specifically, pseudo-anonymization was used where participants were given pseudonyms and identifying details were changed. One week before the data collection began, the study participants were provided with an information sheet and the chance to ask any questions. The aim of the study was repeated in the interviews and the study participants were again given the opportunity to ask questions. After the participants were fully informed about the study, they were required to give written consent for their participation. The manuscript detailing this study’s findings follows O’Brien’s [37] guidelines for qualitative research reporting.

## Results

Several themes and sub-themes emerged from the analysis of the data collected from the diaries and interviews. The four central themes identified are taste, convenience, health concerns, and cultural traditions and rituals.

### Taste

The key role played by taste as a driver of fast food consumption was revealed by the qualitative analysis. In their responses, the participants highlighted the importance of the enjoyable taste of fast food in their decisions to consume this food type. Additionally, they stated that the consistency in the satisfying taste of food from fast food restaurants was important to them. As one participant (P 6) explained:

*“What makes us choose fast food over homemade meals is the taste. For me*,* it doesn’t matter what the food is exactly*,* if it falls under the label of fast food*,* particularly fried food*,* then it will be tasty.” (P 6)*.

This shows that taste is a vital component of the participants’ decision-making regarding fast food. In particular, it highlights the enjoyable taste experience of eating fried foods. A different participant (P 11) discussed the taste of fast food in the following terms:

*“We tried eating at other restaurants*,* but it didn’t work*,* the food was not tasty*,* or if it was tasty once*,* the next time we visited it wasn’t; we didn’t want to risk it again. However*,* the food from popular fast food restaurants tastes good*,* and [these restaurants] will never disappoint us; the taste of their food remains the same over time.” (P 11)*.

The above responses emphasize the importance of not just the enjoyable taste of fast food in driving its consumption but also the consistency of this taste across different visits to fast food restaurants.

### Convenience

The qualitative analysis reveals that savings in time, effort, and money are key contributing factors of the consumption of fast food. This theme shows that people find fast food to be a convenient way of getting their meals. The participants discussed that consuming fast food saves them time, decreases the amount of effort they need to dedicate to meals (by removing the need to purchase and cook ingredients), and is a relatively inexpensive meal option given fast food’s typically low price and the existence of loyalty programs.

#### Time-saving

The sub-theme of “time-saving” reflects the data on how eating fast food can save the participants time and how this contributes to their fast food consumption. The participants discussed how non-fast food options can be time-consuming while fast food is always quick, leaving them more time for other activities. For example, one participant (P 8) stated:

*“It’s all about the time factor. We finish our clinical training at 12 pm*,* and after that we clean and sterilize the clinic*,* so we only have a 40-minute break*,* so*,* you know*,* fast food restaurants are so close and provide meals so quickly*,* then we can return for our 1 pm lecture. If we have a break of several hours*,* maybe we will think of other restaurants. So*,* what drives us to go to fast food restaurants is the time factor.” (P 8)*.

Clearly, the participants’ busy schedules and short break times make time-saving fast food an attractive option. By going to one of the fast food restaurants that are easily accessible in the area around their university, this participant (P 8) can get a meal quickly and return to their lectures and other duties on time.

#### Effort-saving

The sub-theme of “effort-saving” highlights the importance of the ease of fast food meals. According to the participants, eating fast food means that they do not need to prepare and cook meals, saving them a great deal of effort. Similar to fast food’s time-saving characteristic, people with busy lives can minimize the effort they expend on meals by eating fast food and dedicating this effort to other activities. As one participant (P 2) explained:

*“I go to fast food restaurants rather than cooking myself. It is much easier to eat fast food as cooking food is a huge effort for me*,* and you need to wash the dishes afterward. [Eating meals] is somehow tiring if you cook*,* and beyond that*,* we need to get back to our lecture after the break; we have college.” (P 2)*.

For this participant (P 2), fast food represents a huge saving in terms of effort. They prefer to visit a fast food restaurant rather than cook because there is no need to prepare the meal and clean up afterward, leaving them free to quickly return to college.

#### Money-saving

The sub-theme of “money-saving” captures the cost savings that can be made when someone chooses to consume fast food. The participants discussed two key elements in relation to cost savings: the low price of fast food and the loyalty programs (sometimes called “points systems”) that reduce the cost of fast food even more. Through loyalty programs, consumers collect points that they can then use to get discounts, leading to greater savings over time. One participant (P 1) described this factor in the following way:

*“What really drives me to go to fast food restaurants is the saving points systems they offer. I eat at the restaurants at the beginning of each month using my university monthly bonus*,* then I use the points I get to eat free at the end of the month*,* so I don’t spend money and it doesn’t cost me at the end of the month.”* (P 1).

This participant (P 1) explained how they use the loyalty programs offered by fast food restaurants strategically to save money. They eat at a restaurant at the beginning of the month, earn points, and then redeem these points to eat for free or for less at the end of the month. Another participant (P 10) confirmed the generally already low prices of fast food:

*“These [fast food meals] are usually cheap. They are cheaper than other meals.”* (P 10).

Thus, even without the loyalty program discounts, fast food is seen as an inexpensive meal option. The money-saving sub-theme highlights the financial benefits that people believe fast food offers.

### Health concerns

A further core theme identified in the analysis is the impact that people’s concerns about their health is having on their consumption of fast food. The participants revealed that they are becoming increasingly aware of the possible negative health effects of eating fast food and this has prompted them to rethink how much they eat. Talking with their social groups and the growing focus on health and fitness in wider society are both encouraging this increased health awareness. One participant (P 12) summed up this point in the following terms:

*“Recently*,* people around me have started to advise me to take care of my health*,* to try to go to the gym and stuff like that*,* to try to eat healthy food leaving aside fast food and work on my health; people around me keep saying that. You know*,* I am getting older and this will affect my health*,* and I might also get fatter. To be honest*,* I’ve started to think about it. I think this year*,* I will make a plan to eat healthy food and leave fast food alone*,* that is what I’m thinking.” (P 12)*.

This participant explains that their social group has given them a lot of advice about eating healthier and not eating fast food. This has, in turn, prompted them to develop some concerns about their health and to reconsider their consumption of fast food as part of a new, healthier lifestyle. This idea was repeated by other participants in the study, for example, by P 1:

*“This year*,* I will try to adopt a healthier lifestyle; for example*,* when I go to a fast food restaurant*,* I will order less and replace the soda with a juice. I have developed new concerns about my health. Before*,* I would eat anything as long as it was tasty; the only thing that mattered was the taste. Now*,* I’ve started to understand about calories and I have new ideas about what I eat. I have some concerns about my health now.” (P 1)*.

Again, this participant (P 1) discusses the concerns they have recently developed about their health and how these concerns are affecting their consumption of fast food. They highlight the importance of health in their decision-making around fast food and explain that they want to make healthier choices by ordering less at fast food restaurants and drinking juice rather than soda. The “health concerns” theme emphasizes the importance of health perceptions in the participants’ decisions on whether to eat fast food.

### Cultural traditions and rituals

“Cultural traditions and rituals” was the final core theme identified by the qualitative analysis. From this theme, it can be seen that cultural traditions and rituals limit the consumption of fast food for the participants as they do not form any part of the meals eaten in various cultural contexts. Family meals, for example, are described by the participants as being profoundly culturally important and a time to connect with and show respect to family. In this context, homemade and traditional meals are valued, excluding fast food. As one participant (P 19) explained:

*“Usually in our family*,* we are used to the idea that my mum cooks the lunch*,* sometimes edam or rice*,* and you have to eat it*,* you know*,* Mum will get upset if you leave this food and eat outside the home. It’s not acceptable to eat fast food. You know*,* she will wake up early that day to prepare the food for us and she makes an effort*,* particularly since we are a large family. If we go somewhere else*,* she will get upset; all of us want to please her.” (P 19)*.

For this participant (P 19), fast food does not form any part of family meals. Rather, family meals are a time to eat at home and, in particular, to enjoy the food your mother has gone to the effort of preparing for you. Eating outside the home or bringing in fast food would not be acceptable as it undermines the experience of sharing a home-cooked meal as a family.

Other participants also discussed social commitments, special events, and traditional meals as examples of situations where fast food is absent. This is because these contexts typically involve culturally significant, traditional meals. For example, one participant (P 14) said:

*“[This is the case] on Fridays*,* as I said. So*,* with social commitments with close family and relatives*,* we usually gather in certain places outside our homes but we do not eat fast food at all; we go through our rituals*,* usually cooking lamb and rice.” (P 14)*.

As this participant explains, fast food is not consumed at family gatherings. Rather, traditional meals are prepared and eaten. Something similar can be observed during Ramadan, a traditional month of fasting in the Islamic calendar, as discussed by this participant (P 17):

*“Ramadan involves almost no fast food. We eat Ramadan meals*,* soup*,* and stuff like that*,* homemade meals. That is a well-known part [of our culture]. I never eat fast food during Ramadan. That’s normal for us.” (P 17)*.

Ramadan is an important religious festival for many in the KSA and beyond. The participants talked about how fasting during Ramadan directed their food choices. In particular, fast food was not seen as appropriate to eat in this holy month and they preferred to eat more traditional meals. This theme clearly shows that cultural traditions and rituals can have a substantial impact on people’s fast food consumption.

## Discussion

From the literature review, it appears that this study is the first qualitative research in the KSA to examine the contributing factors of fast food consumption in the daily lives of Saudi Arabian university students. The analysis identified the four core themes of taste, convenience, health concerns, and cultural traditions and rituals. According to the results of the analysis, taste is a key driver of fast food consumption. In terms of convenience, the analysis reveals that fast food is perceived to save people time, effort, and money, which drives higher levels of consumption. Fast food consumption was also found to be impacted by health concerns. The sources of these health concerns appear to be the narratives about fitness and health shared in social groups and wider society, which are prompting people to reconsider their food choices. Finally, cultural traditions and rituals such as family gatherings and religious festivals were found to impact fast food consumption as fast food is not a part of mealtimes in these contexts.

The factors linked to the consumption of fast food have been explored in a range of quantitative studies [17, 18, 26, 27, 38–40] and some qualitative research [20–25]. This research confirms previous findings [19–21] by emphasizing the importance of taste as a contributing factor of fast food consumption. The existing research [19] shows that the enjoyable taste of fast food is a significant draw for people, with the majority of participants in one study [20] explicitly identifying taste as a reason they ate fast food. This study builds on these findings by revealing a key new insight: it is not simply the enjoyable taste of fast food that consumers enjoy but the consistency of this taste at subsequent visits to fast food restaurants. Additionally, the fried element of some fast food was identified as especially attractive in terms of taste. These additional details about the importance of taste in driving fast food consumption were uncovered thanks to the data collection methodology applied in this research. Carefully designed semi-structured interview questions and diary prompts allowed us to collect more detailed data compared with that gathered by previous quantitative studies.

The present research also reveals striking evidence for the argument that the perceived financial benefit of eating fast food drives its consumption, as argued in the existing literature [19–23]. Existing research [19] has shown that the low cost of fast food drives its consumption, with one study [20] highlighting the importance of low prices and promotional offers for consumers. This work confirms these findings and broadens our understanding of this area by elaborating upon what “promotional offer” means, that is, it includes loyalty programs where points are received for purchases early in the month, resulting in discounts that drive fast food consumption later in the month. The open-ended nature of our interview questions allowed us to dive deeper and capture these additional details.

According to this study, convenience, or savings in terms of time, effort, and money, contributes substantially to fast food consumption. Similar findings have repeatedly been reported in the literature [17, 20–22, 26, 27], but it is worth noting that “convenience” as a concept has been understood differently in different studies. For instance, some studies [17] use the term “convenience” while others [20] discuss the issue of availability, an aspect that is considered to fall under the umbrella of convenience in the present research. Availability, that is, being able to access fast food easily, was raised as an element of convenience by the study participants in reflections such as “If it’s available around me, I’ll choose a fast food restaurant as it’s convenient for me.” Differences in the understanding of the concept of convenience are due not only to the use of different terminology by researchers but also to the diverse analytical approaches they have taken. To develop a clearer picture of convenience and how it affects the consumption of fast food, this study separates the theme of convenience into three sub-themes: time-saving, effort-saving, and money-saving. Here, “time-saving” refers to the speed with which fast food is prepared and served, relieving people of the need to prepare and cook meals themselves and giving them more time for other tasks. “Effort-saving” refers to fast food’s ability to free consumers from the need to buy and prepare ingredients and clean up after cooking and eating. Finally, “money-saving” concerns the inexpensiveness of fast food in relation to other meal options. Dividing convenience into three parts enhances the discussion on convenience and its impact on fast food and allows this work to be directly compared to existing studies that focus on different aspects of convenience.

The association between health concerns and fast food consumption has also previously been explored [27, 41] by researchers. The participants of Thompson et al.’s study [41] raised the issue of health concerns in their attitudes to fast food and recognized fast food’s potentially adverse impact on their health. This study confirms this finding and also finds that people’s social circles and narratives about health and fitness in wider society are prompting people to think again about how much fast food they consume. This may be due to the growing emphasis that contemporary society is placing on pursuing healthy lifestyles, including making better food choices. To elaborate, an emphasis on healthy living can attach meanings to food that may help shape people’s dietary habits and could be a factor driving a reduction in fast food consumption. This idea of the “meanings” of fast food aligns with previous research [42–44], which states that the meanings attached to things are essential to developing new practices.

The existing literature also discusses the influence of our environment and surroundings on our behaviors [45, 46]. This discussion could exaplain why individuals continue to consume fast food and why they are unable to stop their consumption, as some specific environments can facilitate and encourage the consumption of fast food.

Similarly, a large body of research [19, 21, 25] explores culture’s impact on the consumption of fast food. This study finds that fast food is absent in traditional and cultural contexts, such as family gatherings. The participants highlighted the cultural importance of such meals and of eating the meals carefully prepared by their mothers. According to Warde [47], the act of eating and food more generally can be defined by the location and time of meals and who you eat with; for example, having brunch at a restaurant versus eating Christmas dinner at home with your family. Culture thus influences people’s dietary habits. For the participants in this study, family meals were seen as similar to eating a home-cooked meal with family at Christmas. Nonetheless, research [19] has also found that families eat together in fast food establishments, undermining this finding. These differences in eating habits may be due to cultural differences with some cultures putting a greater value on spending time with family at home.

### Study implications

The results reported in this research have some significant implications for the design of health interventions intended to encourage healthy eating. In particular, these findings suggest that people’s taste preferences should be considered when healthier alternatives are offered and these alternatives should have both an appealing and consistent taste.

Additionally, public health initiatives can concentrate on decreasing the cost and increasing the convenience of healthier food options so that they can compete with fast food and promotional offers for health foods can be introduced.

Moreover, the discussion of health and fitness in wider society appears to be encouraging people to limit their consumption of fast food, and thus this discussion should be nurtured. For example, social media can be used to educate people about healthy eating and increase their awareness of healthy foods as a means of improving public health.

This study also highlights the importance of adopting culturally appropriate approaches to healthy eating strategies. The cultural importance of traditional meals and family mealtimes should be considered by public health workers. Healthier options can be promoted within cultural contexts by collaborating with local communities. Such an approach can conserve the cultural importance of home cooking and encourage healthy eating. Interweaving cultural considerations into public health interventions will increase the effectiveness of these interventions and show appropriate respect for traditional practices. Finally, it is important to educate students about how to avoid fast food consumption and maintain a healthy lifestyle through good dietary choices.

### Study limitations

This research suffers from three key limitations. Firstly, data was gathered through diary entries and semi-structured interviews, which offered useful insights. However, the addition of direct observation would have further enhanced the data collected. Additional data about the contexts and the factors associated with the consumption of fast food would be gained through direct observations conducted in real-world settings. Secondly, this research did not explicitly explore the participants’ preferences regarding healthier alternatives to fast food. By improving our understanding of people’s desires around healthier food and alternative food choices, we could better comprehend what is motivating their food choices, offering policymakers and public health practitioners useful guidance. Thirdly, given the qualitative nature of this research, it surveyed a limited number of study participants. The study population is thus not representative of the broader population and the generalizability of the findings is, therefore, questionable.

### Future research

Subsequent studies could benefit from exploring people’s perceptions of more sustainable and healthier dietary choices as substitutes for fast food. Factors such as people’s attitudes to homemade meals and organic and fresh food options or other types of cuisines with high nutritional value could be examined. By better understanding personal desires and preferences concerning healthier food options, we can gain insights that will help us craft more effective ways to promote healthy eating and design better health interventions to reduce the public’s consumption of fast food.

## Conclusion

The present qualitative study reveals useful information about the factors linked to fast food consumption, namely, taste, convenience, health concerns, and cultural traditions and rituals. The insights reported here have significant implications for public health campaigns designed to promote healthy eating and decrease the consumption of fast food. By carefully considering these factors, health practitioners and policymakers will be able to create initiatives that reflect the public’s preferences and cultural contexts to deliver better health outcomes.

## Data Availability

The datasets used and/or analyzed during the current study are available from the corresponding author on reasonable request.

## Abbreviation

KSA: Kingdom of Saudi Arabia
